# Hybrid ablation for atrial fibrillation: a systematic review

**DOI:** 10.1007/s10840-016-0183-9

**Published:** 2016-09-09

**Authors:** Mindy Vroomen, Laurent Pison

**Affiliations:** 1 Department of Cardiology, Maastricht University Medical Center, PO Box 5800, Maastricht, The Netherlands; 2 Cardiovascular Research Institute Maastricht, Maastricht, The Netherlands

**Keywords:** Atrial fibrillation, Hybrid ablation, Endocardial-epicardial ablation, Review

## Abstract

**Purpose:**

Hybrid ablation for AF is performed in a growing number of centers. Due to absence of guidelines, operative approaches and perioperative care differ per center. In this review, an overview of findings from published studies on hybrid ablations is given, and related topics are discussed (e.g., one- and two-stage approaches, lesion sets, and patient management).

**Methods:**

A systematic literature search was performed in the PubMed and Embase databases. All identified articles were screened and checked for eligibility by the two authors.

**Results:**

Twelve studies describing a total of 563 patients were selected. Due to substantial differences in approaches (one-stage, two-stage, sequential), surgical techniques (bilateral or monolateral thoracoscopy, subxiphoideal, transabdominal), energy sources (unipolar, bipolar), lesion sets (applying left or right atrial lesions), periprocedural care and endpoints (monitoring, definition of recurrence), and success rates (sinus rhythm after a mean of 26 months) are difficult to compare and varied from 27 % (without antiarrhythmic drugs, AADs) to 94 % (with AADs). For studies using bipolar devices, success rates with the use of antiarrhythmic drugs were at least 71 %. Major complications such as bleeding, sternotomy, and death occurred in 7 % of the total population (of which ten complications, 16 %, occurred in the concomitant cardiac surgery hybrid group).

**Conclusion:**

The field of AF ablation has dramatically changed over the past years, with one of the most recent developments the hybrid AF ablation. Lack of matching data hinders drawing conclusions and creating guidelines. Early results however are encouraging. More data are awaiting and needed.

## Introduction

Atrial fibrillation (AF) is the most common cardiac arrhythmia with a lifetime risk of 25 % for people aged above 40 years. Due to an aging population, the incidence is increasing even further [1]. Secondary to negative hemodynamic effects, AF carries significant morbidity and mortality; stroke is the most feared complication with a fivefold increased risk [2]. Therefore, treatment of AF is crucial and worthwhile.

Initially, rhythm control treatment of AF was limited to a direct current shock or taking quinidine, and digitalis was recommended for rate control [3, 4]. In 1982, the first catheter ablation (CA) aimed at achieving rate control by ablation of the atrioventricular junction [5, 6]. The first successful cut and sew surgical treatment (cut and sew Maze procedure) was performed in 1987 by Dr. Cox [7]. As a result of increased knowledge on AF, these procedures have changed extensively over the years. Haissaguerre et al. recognized pulmonary vein (PV) foci as initiators of AF, which currently forms the cornerstone of most interventional treatments for AF [8].

According to current guidelines, pharmacological treatment is still considered as the first step in the approach of AF treatment. However, invasive strategies are gaining more attention. In selected cases, CA [9], or even surgical ablation [10, 11], could be considered as first-line therapy. Although pulmonary vein isolation (PVI) is the cornerstone of AF treatment, no uniform invasive treatment concept in the setting of the nonparoxysmal AF forms exists. Due to suboptimal results of both CA [12] and minimal invasive surgical strategies in this difficult to treat group [13], surgeons and electrophysiologists have combined strengths in the form of a hybrid AF ablation (combination of endocardial catheter and surgical epicardial ablation) to maximize success rates and minimize procedural morbidities. Some ablation lesions associated with the Cox-Maze, for example, cannot be performed minimally invasive, though can be easily performed endocardially. This hybrid approach, first described by Pak et al. [14] in 2007, has already proved to be safe and effective and showed good results in patients suffering from all types of AF [15]. Available data however is still scarce.

Hybrid ablation has now been carried out since a few years and is applied more and more across the world. Absence of guidelines on this procedure leads to the use of different approaches and different insights with respect to patient management. This review aims to provide an overview of findings from published studies on hybrid ablation and discusses related topics (e.g., one- and two-stage approaches, lesion sets, and patient management).

## Methods

The authors have ascribed to the PRISMA standards for systematic reviews [16]. A systematic literature search was conducted in the PubMed and Embase databases. Both structured MeSH terms and free terms were used in the PubMed search. In the Embase search, keywords and free terms were used. The terms are used in such a way that any description that could resemble or relate to hybrid ablation would be covered by the search. Table 1 provides an overview of the search terms. The search was supplemented by manually searching reference lists of selected articles.

**Table 1:** Literature search

Search type	Terms used	Hits^a^ PubMed	Hits^a^ Embase
MeSH terms and Free terms	(“Atrial fibrillation”[MeSH] OR “Atrial fibrillation”[All Fields]) AND ((“hybrid ablation”[All Fields]) OR (“hybrid procedure”[All Fields]) OR (“hybrid approach”[All Fields]) OR (“hybrid”[All Fields]) OR (“endocardial-epicardial ablation”[All Fields]) OR (“surgical-electrophysiological approach”[All Fields]) OR (“thoracoscopic-transcatheter approach”[All Fields]))	210	–
Keywords and Free terms	(Atrial fibrillation) AND (hybrid ablation OR hybrid procedure OR hybrid approach OR endocardial-epicardial ablation OR surgical-electrophysiological approach OR thoracoscopic-transcatheter approach)	–	127

^a^Last search conducted on January 7, 2016

Article inclusion resulted from a three-phase process that consisted of the initial literature search, screening of the literature resulting from the search, and evaluation of eligibility of the articles provided by the screening. Only English literature was included. No publication date or publication status restrictions were imposed.

All articles reporting on hybrid ablation (i.e., more than only a short description of what a hybrid ablation is) were found eligible. First, titles and abstracts were screened. In case of uncertainty, full-text reports were read to assess eligibility. Reference lists of the selected articles were also checked based on the aforementioned criteria. The two authors individually conducted the article search, screening, and selection. In case of disagreement regarding inclusion or exclusion, a paper was discussed to establish consensus.

## Results

### Search results

**Fig. 1:**
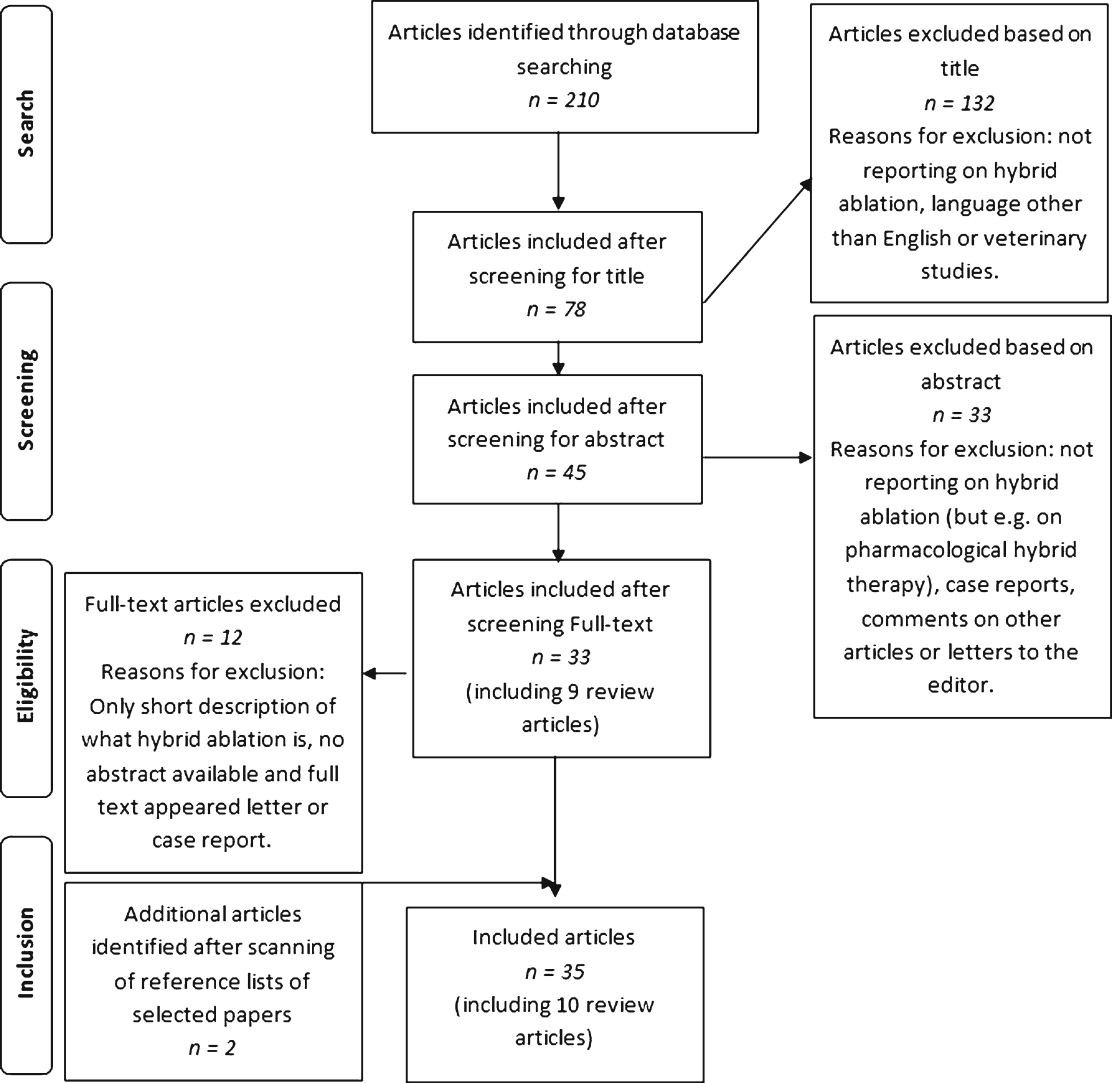
Flowchart of systematic literature search in PubMed and study selection

Following the literature search, 35 papers were included for this review, including 10 review articles [17–26]. No additional papers could be found in the Embase database. In Fig. 1, a flow diagram of the article selection is depicted.

Only 16 of the 35 selected articles describe nonoverlapping original research. The studies of Bulava et al. [27] and Kurfirst et al. [28] are considered overlapping; of these two, Bulava et al. comprises the most recent study and encloses the biggest population. The same accounts for two articles written by La Meir et al. [29, 30], one by Kumar et al. [31], and one by Pison et al. [32], compared to a larger study by Pison et al. [15]. Two articles published by Muneretto et al. [33, 34] are also considered to report on the same patient population. Of these 16 articles, four more are excluded for the main table of this review (Table 2). The article of Kumar et al. [44] was excluded because it consists of only seven patients of which a part of the PVs are treated just epicardial and a part just endocardial with cryoenergy. In the study by R. Lee et al. [45], the electrophysiology (EP) procedure was only performed after the surgical ablation in case of a recurrence (*n* = 7, 29 %), which actually is not a hybrid ablation according to the definition in the guidelines [9]. H.M. Lee et al. [46] operated eight patients and describes a maximum follow-up period of only 6 months, and Velagic et al. [47] mainly concentrates on the redo procedures after a hybrid ablation.

**Table 2:** List of studies on hybrid AF ablation

First author	Patients (number)	AF duration (years)	LAD (mm)	(ls)pAF (%)	FU (months)	FU reached (%)	Monitoring	SR (%) +/− AAD
Bisleri [35]	45	7 ± 6	51 ± 10	100	28 ± 2	100	ILR	89/–
Bulava [27]	50	4 ± 3	48 ± 4	100	17 ± 5	100	7-day	94/84
Gehi [36]	101	6 ± 6	51 ± 10	83	12	100	24-h	66/37
Gersak [37]	50^b^ 45	5 ± 5	48 ± 1	94	12	70	ILR	88/75
Krul [38]	31	8 (1-25)	47 ± 7	48	12	71	24-h	–/86
La Meir [39]	19	5 (3–8.5)	49 ± 20	74	12	100	7-day	63/37
Mahapatra [40]	15	5 ± 1	52 ± 10	100	16 ± 2	100	24-h	93/87
Muneretto [34]	36	6 (0.5–20)	50 ± 6	100	30 (1–58)	100	ILR	92/78
Pison [15]	78	4 (2–7)	45 (42–48)	63	24 (12–36)	100	7-day	86/74
Richardson [41]	83	–	49 (42–53)	99	12	95	ILR	71/61
Zembala [42]	27	4 ± 3	46 ± 5	82	12	37	7-day or ILR	0/80
Total	535^b^	–	–	–	–	–	–	–
Mean		5	49	86	17	89	–	82/70
Gaita [43]^a^	33	2 ± 2	51 ± 6	100	128 ± 37	100	Holter	33/27
Total	568^b^	–	–	–	–	–	–	–
Mean		5	49	87	26	89	–	78/66

*AF* atrial fibrillation, *LAD* left atrial diameter, *(ls)pAF* long-standing persistent AF, *FU* follow-up, *SR +/- AAD* sinus rhythm with/without antiarrhythmic drugs, *ILR* implantable loop recorder, *7-day* 7-day holter monitoring, *24-h* 24-h holter monitoring, *holter* not stated which is used

^a^Open concomitant hybrid surgery

^b^Including five patients with only epicardial ablation (not separated in baseline table in article)

Following this final selection, the main characteristics of the 12 remaining studies are presented in Table 2. The studies have all been published between 2011 and January 2016. The mean age of the patients over all studies is 60 years (ranging from 53 to 63), and 71 % is male (*n* = 405). Some articles reported follow-up at different moments in the same study; in Table 2, the follow-up moment with the largest patient population is mentioned or, when the difference in population was only little, the moment with the longest follow-up is taken. In case of use of different monitoring types, the one with the longest recording time is mentioned.

### Patient selection

One of the most important issues in hybrid ablation is patient selection. According to the guidelines, a minimally invasive surgical ablation or a hybrid ablation can be performed (as stand-alone procedures) in patients with symptomatic AF after failure of CA, or in patients who prefer a surgical/hybrid approach [9]. In 2012, a European survey showed that failure of CA was the most important indication for performing a stand-alone invasive procedure [48]. However, looking at the publications on hybrid ablation, this procedure mostly is not only applied in cases of failed CA. In the study of Gehi et al. [36], only 36 out of 101 patients underwent a previous CA, Zembala et al. [42] reported 8 out of 27 patients with previous CA, Krul et al. [38] 14 out of 31, La Meir et al. [39] 9 out of 19, and Pison et al. [15] 39 out of 78. Only Mahapatra et al. [40] performed hybrid ablation solely in patients with a previous CA. Pison et al. [15] referred patients for a hybrid procedure in cases of paroxysmal, persistent, or long-standing persistent AF (PAF, pAF, lspAF) with a left atrial (LA) volume index ≥29 mL/m^2^, after one or more failed CA or based on patient preference. Gehi et al. [36] considered a hybrid procedure if the patients were expected to have a less efficient outcome of a standard CA, which included patients after failed CA with concomitant antiarrhythmic drugs (AADs), patients with pAF with large LA size (not stated what is considered large) or structural heart disease, and lspAF patients. Another indication for a hybrid ablation, which is not mentioned in the selected articles, could have been a thrombo-embolic advantage with left atrial appendage (LAA) exclusion. The indications for a stand-alone hybrid procedure are thus expanding and are expected to increase in the future.

Besides having clearly outlined indications, it is also important to be able to predict outcome. P-wave duration has shown to be a marker of success of AF ablation procedures and could be of help in the selection. Kumar et al. [49] found that the P-wave duration immediately and significantly shortened after a hybrid procedure, in both PAF and pAF patients. Pre-procedural duration was found to be longer in pAF and lspAF, than PAF patients; post-procedural, no difference existed. The pre- versus post-procedural P-wave duration was significant for patients without recurrence. This suggests that patient selection based on type of AF may be possible before the procedure, which enables individualization of therapy. Besides P-wave duration, clearly type and duration of AF, and LA diameter are predictors for the outcome, as well as the presence of multiple risk factors like diabetes mellitus and hypertension [19, 50].

### One- or two-stage approach

The different options to perform a hybrid procedure are a one-stage approach (surgery and EP during the same procedure), a sequential approach (EP later but during the same hospital admission), and a staged approach (EP in a second hospital admission, maximum 6 months after surgery). Muneretto et al. [34] and Bisleri et al. [35] performed the EP 30–45 days after the surgical ablation, Bulava et al. [27] 6 to 8 weeks later, and Gaita et al. [43] after the blanking period of 3 months. In two studies [40, 46], a sequential approach was performed, meaning EP circa 4 days after the surgical ablation (3 % of the patients). Zembala et al. [42] first performed one-stage procedures but changed (due to reimbursement issues) to a two-stage approach with a second admission 15–20 days after the surgical ablation. Richardson et al. [41] and Gersak et al. [37] performed both, in which the approach depended on operator preferences in the first study. The staged approach was used in 41 % of the patients (*n* = 232). Almost all patients with a staged approach underwent the planned second step (97 %). Of 195 patients was reported whether additional endocardial ablation was necessary, this was the case in 56 % (*n* = 110). All other studies [15, 36, 38, 39] described a one-stage procedure (*n* = 321, 56 %).

### Surgical approach

Fundamentally all hybrid procedures are minimally invasive; however, the surgical approach itself can vary considerably. Five groups used a bilateral thoracic approach [27, 38, 40, 41, 46], three groups used a right thoracic monolateral approach [34, 35, 39], one used a subxiphoideal approach [42], and two others used a transabdominal transdiaphragmatic approach [36, 37]. The group of Pison et al. [15, 23, 32] used bilateral, left and right monolateral thoracoscopic approaches. Gaita et al. [43] was the only group to perform open hybrid surgery, combined with valve or coronary surgery.

### Ablation techniques

Surgical epicardial ablation can be performed with cryoenergy, unipolar or bipolar radiofrequency energy (RF), endocardial ablation with microwave energy, cryoenergy, and unipolar RF. Bipolar RF was used epicardially in six series [15, 27, 38, 40, 41, 46], which equals the reported use of unipolar RF [34–37, 39, 42]. Gaita et al. [43] used cryoenergy in their open hybrid procedure, but Kumar et al. [44] also used cryoenergy in rare cases thoracoscopically. In the latter, in a cohort of seven patients with Gold Class IV chronic obstructive pulmonary disease (COPD), the right PVs were isolated using epicardial bipolar RF (Atricure) and the left PVs were isolated using an endocardial 28-mm cryoballoon.

### Lesion sets

As expected, with the PVs being well-known foci for developing AF, PVI was performed epicardially in all patients in all studies. Additionally, performing an epicardial roof (connecting line between the superior PVs) and inferior line (connecting line between the inferior PVs), i.e., a box lesion, is the most performed lesion set [15, 27, 34–36, 39, 41, 42]. Three papers only mention performing a roof line but not an inferior line [37, 40, 43]. Krul et al. [38, 51] created roof lines in case of pAF and lspAF, and only in selected cases also an additional inferior line.

Besides PV with roof and inferior lines, some alternative epicardial linear lesions can be created. These comprise a line from the roof line to the left trigone, a line from the superior PV to the LAA, a line from the RIPV to the coronary sinus (CS), a superior vena cava (SVC) and inferior vena cava (IVC) circumferential lesion, and an intercaval line (line between SVC and IVC). The right atrial lesions are less frequently used than the left atrial lesions. Whether or not these lesions were made, differed per patient. The intercaval line is only reported by Pison et al. [15] and Richardson et al. [41], which in case of Pison et al. was only added to the treatment in case of pAF or lspAF in combination with a dilated right atrium (>58 mL). Lee et al. [46] also performed ablation of ganglionated plexi and the ligament of Marshall. The latter was also performed by Richardson et al. [41]

The endocardial lines are less described than the epicardial lines. Reported lines are left mitral isthmus (MI) line (from the inferior margin of the LIPV ostium to the mitral annulus), cavotricuspid isthmus (CTI) lines (between the IVC and the tricuspid annulus), and CS line (distal CS to the os). In the published studies, performing these endocardial lines strongly depended on the presence of arrhythmias like atrial flutters, and on the preference of the cardiologist. Also, complex fractionated atrial electrograms (CFAE) ablation are described. The MI and CTI lines are most performed [15, 27, 35, 36, 40, 41].

### Left atrial appendage

The LAA is deemed to be the origin for more than 90 % of emboli in nonvalvular AF [52]. Despite its proven efficacy, oral anticoagulation (OAC) therapy can lead to bleedings and may be difficult to control in many patients. Therefore nonpharmacological treatment strategies to reduce the risk of stroke in AF patients have been developed, including LAA occlusion or removal during rhythm surgery. Bisleri et al. [35] and Gaita et al. [43] do not describe LAA therapy. Three studies did not perform a thoracoscopic ablation, leading to impossibility to address the LAA and necessity for OAC after the ablation [36, 37, 42]. In cases of resternotomy in these studies, the LAA was removed but no motivation for removal was reported. Muneretto et al. [34] “avoided LAA treatment” (cited), since at that time, no safe and validated techniques for exclusion of the LAA via a right thoracic approach existed. In three studies [38, 40, 41, 46], the LAA was removed (using a stapler or clip) in every patient but they did not substantiate the reasoning. Bulava et al. [27] excluded the LAA whenever deemed safe and feasible by the surgeon, which was the case in 84 % of the patients.

The only groups giving clear argumentation for LAA treatment were Pison et al. and La Meir et al. [15]: “in patients with a CHA2DS2-VASC ≥1, or in the presence of a rapid firing coming from the LAA, and when the procedure was deemed safe, LAA exclusion/closure was performed under transoesophageal echocardiographic (TEE) guidance employing a stapler or a clip.” They excluded/closed the LAA in 35 patients, which is 44.9 %.

In none of the articles is spoken about TEE performed in the follow-up to check LAA closure, so in this population, no data is available on long-term occlusion and thromboembolic processes in the LAA. Neither is information available on what influence LAA exclusion/closure has on AF recurrence. It could, however, be that LAA exclusion/closure positively influences the results [53, 54].

### Periprocedural care

The main topics discussed in the literature on periprocedural care are use of AADs and regulation of OAC. Mahapatra et al. [40] maintained the patients on warfarin for at least 1 month prior to the hybrid procedure and stopped 5 days before. Except for amiodarone, AADs were stopped for five half-lives. Krul et al. [38] discontinued OAC 3 to 4 days prior to the procedure, and AADs were continued during admission. Bulava et al. [27] stopped OAC 7 days before the hybrid procedure, after which a low-molecular-weight heparin (LWMH) was started until the evening before the procedure. Other articles not clearly describe preoperative management.

After the procedure, Krul et al. [38] transferred the patients to the ward, where coumarin derivates were restarted before removal of the chest drains the day after the procedure. Unfractionated heparin was started as soon as bleeding risk allowed this and was continued until an INR of 2.0 was reached. OAC was discontinued after 6 months. Muneretto et al. [34] and Bisleri et al. [35] neither transported patients to the intensive care unit (ICU), where in three other studies [27, 42, 46], all patients were transported to the ICU. The latter administered LMWH 6 h after surgery and restarted warfarin after removal of thoracic drains. Five authors [15, 34, 37, 39, 40] gave AADs postoperatively to all patients and recommended discontinuation after 3 months if the patient was free of AF. They started warfarin on the second postoperative day (target INR of 2.5) and stopped after 3 months if the patient was in SR or had a CHA_2_DS_2_VASc score of <2. Bulava et al. [27] did not prescribe any AAD at time of discharge. Gehi et al. [36] prescribed OAC and an AAD for at least 6 weeks, Lee et al. [46] for 6 months. Zembala et al. [42] administrated intravenous amiodaron and heparin 1 h after the surgery for 48 h. On day 2, warfarin was started. In case of a staged approach, patients were discharged on LMWH and after EP warfarin was started which was replaced by aspirin after 6 months in case of SR. AADs were continued for 3 months.

### Rhythm monitoring and endpoints

All studies used a long-term monitoring method, either 24-h holter monitoring [38], 7-day holter monitoring [15, 27, 39, 42], or an implantable loop recorder [34, 35, 37, 41, 42]. Two studies did not specify the duration of the holter [43, 46]. Mahapatra et al. [40] used a 7-day continuous autotriggered monitor (CAT) and a 24-h holter. Patients with an implantable loop recorder were instructed to return monthly to have their monitors downloaded and interrogated. Further electrocardiogram visits where performed in all studies, but time spans differed, and patients were instructed to return in case of palpitations.

Success was defined as the absence of AF and/or any other supraventricular arrhythmia lasting more than 30 s on a 7-day holter monitor during the entire follow-up after the blanking period [27, 41, 42], which corresponds with the guidelines [9]. Three other studies included freedom of AADs in the definition of success [15, 39, 40]. In two articles, a 24-h holter was used to confirm freedom of AF [36, 38]. Some considered stable SR as the absence of AF episodes lasting more than 5 min and an overall burden of 0.5 % of time spent in AF on a monthly basis [34, 35]. Gaita et al. [43] considered success if the patient maintained stable SR or suffered from only brief episodes of paroxysmal tachyarrhythmia’s with spontaneous restoration, but also patients requiring cardioversion were considered successful if SR maintained after the cardioversion. Gersak et al. [37] did not describe clear endpoints.

### Procedural results and complications

Success rates (sinus rhythm after a mean of 26 months) without the use of AADs from the studies presented in Table 2 ranged from 27 % [43] to 87 % [40]. Success rates with the use of AADs ranged from 33 % [43] to 94 % [27]. Concerning the studies which included a limit of 30 s in their definition of recurrence, success rates were 61 % [41], 74 % [27], 80 % [42], 84 % [15], and 87 % [40]. In the study of Zembala et al. [42], nobody in SR used AADs anymore. According to the difference between unipolar and bipolar conducted ablations, the mean of the success rates is in favor of the bipolar devices (82.7 vs. 46.9 %). In seven of the articles [15, 27, 36–38, 42, 43], a total of 27 redo procedures are reported. No information is given on findings during these procedures. Velagic et al. [47] does describe findings of redo procedures after hybrid ablation: in 36 % of the 14 patients recovered conduction was found, but only 9 % of the PVs were reconnected and 7 % of the box lesions.

Only three papers report success rates by type of AF. Krul et al. [38] reported, without use of AADs, 91.7 % for PAF (*n* = 12), 77.8 % for pAF (*n* = 9), and 100 % for lspAF (*n* = 1). Pison et al. [15] reported, without AADs, 76 % for PAF (*n* = 22), 62 % for pAF (*n* = 28), and 100 % for lspAF 100 % (*n* = 15), and with AADs 97 % for PAF (*n* = 28) and 85 % for pAF (*n* = 29). La Meir et al. [39] reported 60 % for PAF (*n* = 3), 50 % in pAF (*n* = 2), and 20 % in lspAF (*n* = 2).

Bulava et al. [27] elaborated in their article EP findings in relation to patients in SR and patients in a tachyarrhythmia at the time of EP. Of the patients in SR, 74.4 % of all PVs were isolated (*n* = 29/39). Of the patients with a tachyarrhythmia, this was 72.2 % (*n* = 8/11). In 30 % of all patients (*n* = 15), the box lesion was found to be complete, with no difference in patients in SR versus patients with an arrhythmia. At the end of the EP, 100 % of the lesions were completed. In 42 % of the patients in the study by Gaita et al. [43], the lesion set was found to be incomplete: in 3 patients, the PVs were not completely isolated, in 3 patients the roof line, and in 11 patients the MI line. At the time of EP, 15 of the 33 patients were in SR. After EP, 79 % of the lesions could be completed. Of the ten patients who never had recurrences, nine had a complete lesion set after EP. In the patients where no complete scheme could be achieved, 43 % were in SR, all on AADs, and 57 % in permanent AF. Bisleri et al. [35] found, during EP, in 91.1 % of the patients a complete box lesion and reached 100 % after EP, for Mahapatra et al. [40], this was 83.3 % and also 100 % at the end of the EP. In two articles only is mentioned what is reached at the end of the EP (PVI), 97 % [36] and 100 % [38]. Pison et al. reported a complete box lesion at the time of EP in 64 % of the patients and, after endocardial touch-up, at the end of the EP in 100 %. La Meir et al. [39] found that during EP, none of the patients with a complete box, and 17 out of 19 patients had at least one PV not isolated for which endocardial touch-up was performed.

The periprocedural complications are listed in Table 3. In total, 63 complications occurred (including 23 minor complications, e.g., pneumonia), which concurs with 11 % of the patients. A major complication occurred in 7 % of the patients (of which 16 % in the concomitant cardiac surgery hybrid group) and death in 1.6 %. Three of the deaths occurred in the concomitant group, excluding these gives a mortality rate of 1.1 %.

**Table 3:** Reported complications

First author	Bisleri [35]	Bulava [27]	Gaita [43]	Gehi [36]	Gersak [37]	Krul [38]	Mahapatra [40]	Meir [39]	Muneretto [34]	Pison [15]	Richardson [41]	Zembala [42]	Total
Death	–	–	3	2	2	–	–	–	–	–	1	1	9
Stroke	–	–	–	–	1	–	–	–	–	–	–	–	1
Sternotomy/reoperation	–	2	–	–	–	3	–	–	–	1	–	1	7
Permanent phrenic nerve injury	–	4	–	–	–	–	–	–	–	–	–	–	4
Tamponade/effusion	–	1	–	2	1	–	–	–	–	–	–	1	5
Pacemaker implantation	–	–	5	–	–	–	–	–	–	2	1	–	8
Major bleeding	–	–	2	2	1	1	–	–	–	–	–	–	6
Minor	–	10	–	–	–	3	–	–	–	2	8	–	23
Total	0	17	10	6	5	7	0	0	0	5	10	3	63

Minor complications: postoperative infection, PV narrowing, temporary phrenic nerve injury, pneumothorax, blood transfusion ≤2 units

## Discussion

The main purpose of this review was to provide an overview of findings and experiences from published studies on hybrid ablations and to discuss related topics. The approaches, techniques, lesion sets, periprocedural care, and endpoints substantially differ per center, which makes it difficult to compare results. Success rates (after a mean follow-up of 26 months) without the use of AADs ranged from 27 to 87 %. The very low success rate of Gaita et al. [43] most probably relates to the fact that the population concerns patients with concomitant surgery. La Meir et al. [39] and Gehi et al. [36] also report low success rates; they both used unipolar devices which, compared to bipolar devices, less reliably produce permanent transmural lesions and are less effective in restoration of SR [30, 55]. The failure of these two studies might thus relate to the use of ineffective ablation techniques, though also when using bipolar devices, linear lesions are not always completely transmural and need touch-up during the endocardial part of the hybrid ablation [32], and other articles in which unipolar devices are used showed better results. These however concern a lower number of patients with pAF and lspAF, and lower existence of comorbidities.

The results of the hybrid approach have been compared (nonrandomized) to standard minimally invasive surgical approaches, showing better 1-year results in the hybrid group [29]. In another trial comparing CA to surgical ablation, surgical ablation was found to be superior to CA, although the procedural adverse events were significantly higher for surgical ablation [56]. However, due to some study limitations, success and complication rates might be overestimated.

Another topic to highlight is the surgical approach. The monolateral approach, compared to the bilateral approach, reduces risk of complications as bleeding, pneumothorax, and lung hernia [57, 58] and is supposed to be less painful, improving the patient’s recovery. It is preferred in patients with COPD. Since the heart is enlarged to the left of midline, a right thoracic approach provides additional working space compared to a left-sided approach. However, with a right-sided approach it is more difficult to occlude or exclude the LAA. In case of a left-sided approach, it is possible to isolate the right PVs using the standard bipolar devices, which would not be possible the other way around [23]. To reach the left PVs using a right thoracoscopy, an ablation probe delivering continuous lesions encircling the origin of all PVs and the posterior aspect of the LA all together, creating a box lesion, is needed [34, 35, 39]. It is however doubtful if this probe can create sufficient transmural lesions.

Probably the most discussed issue is whether the hybrid ablation procedure should be performed in one stage, sequential, or in two stages. Each approach has its opportunities but also challenges. It seems that the main concern with a one-step approach is that it is time-consuming for both the surgeon and the electrophysiologist, and therefore, it even has been called a “logistical nightmare.” Furthermore, the procedural environment has to be optimized for both operators. Another described disadvantage is the formation of edema of the myocytes due to damage as result of the ablation, with difficulty to test and reablate the same area with CA. Managing the periprocedural anticoagulation (especially heparinisation after transseptal puncture in combination with operation wounds) can be challenging. CA in a second admission theoretically could enable identification of areas of early reconnection. Further, it allows gaps to develop and to be detectable, since small gaps could have been missed at an initial procedure. Richardson et al. [41] showed that a staged approach increased the likelihood of discovering incomplete lesions; however, this did not improve the time to recurrence. The inconvenience for the patient is significantly lower in a one-stage approach compared to a sequential and two-stage approach with a second hospital admission and a second time anesthesia. Next, complications could be minimized in a one-step approach: there is less risk of phrenic nerve damage or esophageal injury since the surgeon can protect these structures, and with an opened pericardium, the risk of tamponade during transseptal puncture is lowered. A one-stage approach allows for immediate electroanatomic mapping with endocardial confirmation of isolation or incomplete isolation with the possibility to add touch-up lesions endocardially, and it also allows for guiding epicardial ablation, including identifying substrates which may be more effectively targeted epicardially. An argument to choose for a sequential, instead of a two-stage, approach could be abating the risk that patients in SR after the first procedure will not come back for a second procedure, and the inconvenience for the patient will be lower when only admitted once to the hospital. A head to head study comparing the different approaches should be performed to support the discussion on this topic.

The final but perhaps most important question to answer is, why hybrid? And which patients should be advised to undergo a hybrid ablation? There are several advantages of a hybrid ablation. First, it is important to confirm conduction block of the ablation lines, since incomplete lines makes the patient susceptible for developing atrial flutters [59]. Due to immediate mapping, it is also possible to tailor the epicardial lesion set. Next, some lines cannot be accomplished epicardially, however easily endocardially, and it is easier to perform some of the endocardial lesions epicardially (for example, ligament of Marshall). Disadvantages are the prolonged procedure time, heparinization after the surgical procedure and it (possibly unnecessarily) exposes the patients to the risks of both CA and thoracoscopy. The little available research shows that mainly lspAF patients will have advantage of the hybrid procedure compared to the surgical procedure [29], and that patients with previous failed CA have advantage of a hybrid procedure compared to a redo CA procedure [40]. Obviously, more research is needed to be able to answer these questions well-founded and to compare the results of the different ablation strategies. At the moment, some trials are performed and answers are expected in the next years from among others the DEEP-AF (NCT01246466), CONVERGE-AF (NCT01984346), HARTCAP-AF (NCT02441738), and EHAFAR (registry) studies.

## Conclusion

The field of AF ablation has dramatically changed over the past years, with the most recent development a close collaboration between an electrophysiologist and a cardiac surgeon in the form of a hybrid AF ablation. Absence of guidelines on this procedure leaded to various strategies for performing the ablation, making it difficult to report results. Insufficient data are available comparing hybrid ablation to surgical or catheter ablation. This hinders drawing well-founded conclusions about the procedure. Considering the available data, the AAD-free success rates are satisfactory and hybrid ablation is a promising procedure, but more (multicenter, randomized) data are necessary to confirm the early results and give hybrid ablation a definitive position in the guidelines for AF therapy.
